# Supplementary material for: Comparative multiomics analysis of cell physiological state after culture in a basket bioreactor
Source: Sci Rep. 2022 Nov 23;12:20161. doi: 10.1038/s41598-022-24687-4 (PMC9686226; doi:10.1038/s41598-022-24687-4)
Supplement: Supplementary file 1 — Supplementary Information 1. [file 41598_2022_24687_MOESM1_ESM.zip › raw data/Transcriptomic raw data/6.Enrichment/1.GO/TE1vsTC1/GSEA/TE1/PROTEIN_LOCALIZATION/PROTEIN_LOCALIZATION(GO_0008104).html]

Details for gene set PROTEIN\_LOCALIZATION(GO:0008104)[GSEA]


|  || Dataset | readcount.sample |
| Phenotype | sample.cls |
| Upregulated in class | TE1 |
| GeneSet | PROTEIN\_LOCALIZATION(GO:0008104) |
| Enrichment Score (ES) | 0.35777166 |
| Normalized Enrichment Score (NES) | 1.8122768 |
| Nominal p-value | 0.0 |
| FDR q-value | 0.21006808 |
| FWER p-Value | 0.494 |
Table: GSEA Results Summary

  

Fig 1: Enrichment plot: PROTEIN\_LOCALIZATION(GO:0008104)
  
Profile of the Running ES Score & Positions of GeneSet Members on the Rank Ordered List

  

| PROBE | DESCRIPTION   (from dataset) | GENE SYMBOL | GENE\_TITLE | RANK IN GENE LIST | RANK METRIC SCORE | RUNNING ES | CORE ENRICHMENT || 1 | 103222840 | LOC103222840 |  |  | 441 | Infinity | -0.0197 | Yes |
| 2 | 103225893 | LOC103225893 |  |  | 560 | Infinity | -0.0250 | Yes |
| 3 | 103224720 | LOC103224720 |  |  | 680 | 7.298 | 0.0164 | Yes |
| 4 | 103231257 | LOC103231257 |  |  | 1095 | 3.305 | 0.0189 | Yes |
| 5 | 103240797 | LOC103240797 |  |  | 1560 | 2.400 | 0.0134 | Yes |
| 6 | 103236180 | LOC103236180 |  |  | 1731 | 2.265 | 0.0203 | Yes |
| 7 | 103232300 | LOC103232300 |  |  | 1824 | 2.207 | 0.0303 | Yes |
| 8 | 103228869 | SEC23A |  |  | 1967 | 2.101 | 0.0374 | Yes |
| 9 | 103240505 | IPO7 |  |  | 2289 | 1.938 | 0.0353 | Yes |
| 10 | 103229165 | AKAP5 |  |  | 2488 | 1.860 | 0.0384 | Yes |
| 11 | 103239820 | COPB1 |  |  | 2922 | 1.727 | 0.0300 | Yes |
| 12 | 103235715 | SRP72 |  |  | 3021 | 1.701 | 0.0364 | Yes |
| 13 | 103226057 | SEC61G |  |  | 3120 | 1.682 | 0.0428 | Yes |
| 14 | 103246817 | KDELR2 |  |  | 3306 | 1.644 | 0.0450 | Yes |
| 15 | 103247446 | LOC103247446 |  |  | 3369 | 1.633 | 0.0527 | Yes |
| 16 | 103240726 | AKAP12 |  |  | 3572 | 1.596 | 0.0538 | Yes |
| 17 | 119619579 | SRP9 |  |  | 3576 | 1.596 | 0.0639 | Yes |
| 18 | 103230793 | TOMM20 |  |  | 3669 | 1.580 | 0.0699 | Yes |
| 19 | 103220190 | XPO1 |  |  | 3715 | 1.571 | 0.0779 | Yes |
| 20 | 103242823 | CLTC |  |  | 3941 | 1.535 | 0.0776 | Yes |
| 21 | 103237959 | STAM |  |  | 4075 | 1.517 | 0.0814 | Yes |
| 22 | 103240502 | KPNA5 |  |  | 4156 | 1.508 | 0.0874 | Yes |
| 23 | 103244343 | SRP19 |  |  | 4184 | 1.505 | 0.0958 | Yes |
| 24 | 103218923 | LOC103218923 |  |  | 4287 | 1.487 | 0.1008 | Yes |
| 25 | 103241428 | SSR3 |  |  | 4441 | 1.468 | 0.1033 | Yes |
| 26 | 103228813 | SRP54 |  |  | 4484 | 1.462 | 0.1108 | Yes |
| 27 | 103235816 | USO1 |  |  | 4535 | 1.455 | 0.1178 | Yes |
| 28 | 103233628 | SCAMP4 |  |  | 4727 | 1.433 | 0.1184 | Yes |
| 29 | 103223321 | TOMM22 |  |  | 4770 | 1.426 | 0.1257 | Yes |
| 30 | 103245632 | AP4E1 |  |  | 4815 | 1.421 | 0.1328 | Yes |
| 31 | 103234478 | LOC103234478 |  |  | 4938 | 1.408 | 0.1363 | Yes |
| 32 | 103239119 | TSG101 |  |  | 4954 | 1.407 | 0.1446 | Yes |
| 33 | 103217575 | PGAP1 |  |  | 4994 | 1.402 | 0.1518 | Yes |
| 34 | 103219194 | CLTA |  |  | 5081 | 1.392 | 0.1569 | Yes |
| 35 | 103228184 | KPNA1 |  |  | 5210 | 1.378 | 0.1600 | Yes |
| 36 | 103231289 | SELENOS |  |  | 5220 | 1.377 | 0.1684 | Yes |
| 37 | 103244556 | SEC24A |  |  | 5284 | 1.370 | 0.1743 | Yes |
| 38 | 103242897 | TOM1L1 |  |  | 5505 | 1.349 | 0.1730 | Yes |
| 39 | 103227635 | DAG1 |  |  | 5709 | 1.330 | 0.1724 | Yes |
| 40 | 103215432 | XPO7 |  |  | 6061 | 1.296 | 0.1650 | Yes |
| 41 | 103232201 | VPS35 |  |  | 6125 | 1.291 | 0.1704 | Yes |
| 42 | 103222151 | IPO11 |  |  | 6147 | 1.289 | 0.1777 | Yes |
| 43 | 103214430 | KPNA3 |  |  | 6252 | 1.278 | 0.1812 | Yes |
| 44 | 103222967 | TNPO1 |  |  | 6265 | 1.276 | 0.1888 | Yes |
| 45 | 103225204 | KPNA6 |  |  | 6560 | 1.257 | 0.1837 | Yes |
| 46 | 103233854 | NXF1 |  |  | 6631 | 1.249 | 0.1885 | Yes |
| 47 | 103226389 | TOMM7 |  |  | 6643 | 1.248 | 0.1960 | Yes |
| 48 | 103228713 | IPO4 |  |  | 6683 | 1.244 | 0.2022 | Yes |
| 49 | 103221273 | SEC62 |  |  | 6737 | 1.240 | 0.2078 | Yes |
| 50 | 103239129 | UEVLD |  |  | 6798 | 1.236 | 0.2130 | Yes |
| 51 | 103230327 | IPO9 |  |  | 6820 | 1.235 | 0.2199 | Yes |
| 52 | 103245852 | SRP14 |  |  | 6858 | 1.232 | 0.2262 | Yes |
| 53 | 103214377 | COG3 |  |  | 6864 | 1.232 | 0.2338 | Yes |
| 54 | 103234972 | KDELR1 |  |  | 6939 | 1.226 | 0.2383 | Yes |
| 55 | 103236101 | SEC24B |  |  | 6965 | 1.224 | 0.2451 | Yes |
| 56 | 103232989 | BCAP31 |  |  | 6969 | 1.224 | 0.2528 | Yes |
| 57 | 103223509 | AP3B1 |  |  | 7008 | 1.220 | 0.2589 | Yes |
| 58 | 103233183 | IST1 |  |  | 7022 | 1.219 | 0.2661 | Yes |
| 59 | 103235773 | LOC103235773 |  |  | 7075 | 1.215 | 0.2715 | Yes |
| 60 | 103228100 | SEC61A1 |  |  | 7188 | 1.206 | 0.2742 | Yes |
| 61 | 103223316 | KDELR3 |  |  | 7232 | 1.202 | 0.2800 | Yes |
| 62 | 103244420 | SNX2 |  |  | 7239 | 1.201 | 0.2874 | Yes |
| 63 | 103226195 | VPS41 |  |  | 7388 | 1.191 | 0.2883 | Yes |
| 64 | 103235185 | AP2A2 |  |  | 7512 | 1.180 | 0.2904 | Yes |
| 65 | 103230911 | CNST |  |  | 7744 | 1.162 | 0.2874 | Yes |
| 66 | 103214961 | SEC23B |  |  | 7812 | 1.156 | 0.2918 | Yes |
| 67 | 103220202 | PEX13 |  |  | 7877 | 1.153 | 0.2963 | Yes |
| 68 | 103244616 | CSE1L |  |  | 7905 | 1.150 | 0.3025 | Yes |
| 69 | 103243153 | KPNA2 |  |  | 8015 | 1.142 | 0.3049 | Yes |
| 70 | 103226921 | EXOC4 |  |  | 8016 | 1.142 | 0.3122 | Yes |
| 71 | 103242051 | COPB2 |  |  | 8037 | 1.140 | 0.3186 | Yes |
| 72 | 103237891 | SEC61A2 |  |  | 8048 | 1.139 | 0.3254 | Yes |
| 73 | 103226516 | SEM1 |  |  | 8238 | 1.131 | 0.3242 | Yes |
| 74 | 103223741 | MTX3 |  |  | 8565 | 1.113 | 0.3166 | Yes |
| 75 | 103241393 | KPNA4 |  |  | 8569 | 1.113 | 0.3236 | Yes |
| 76 | 103241183 | TGFBRAP1 |  |  | 8943 | 1.089 | 0.3138 | Yes |
| 77 | 103233646 | AP3D1 |  |  | 9032 | 1.084 | 0.3168 | Yes |
| 78 | 103243578 | KPNB1 |  |  | 9047 | 1.083 | 0.3231 | Yes |
| 79 | 103226799 | LOC103226799 |  |  | 9050 | 1.082 | 0.3300 | Yes |
| 80 | 103236172 | SEC24D |  |  | 9057 | 1.082 | 0.3366 | Yes |
| 81 | 103233178 | AP1G1 |  |  | 9161 | 1.075 | 0.3389 | Yes |
| 82 | 103226904 | COPG2 |  |  | 9381 | 1.061 | 0.3358 | Yes |
| 83 | 103245499 | SNX1 |  |  | 9422 | 1.059 | 0.3408 | Yes |
| 84 | 103226712 | BCAP29 |  |  | 9554 | 1.051 | 0.3416 | Yes |
| 85 | 103248787 | SRPRA |  |  | 9555 | 1.051 | 0.3484 | Yes |
| 86 | 103230424 | TPR |  |  | 9717 | 1.040 | 0.3478 | Yes |
| 87 | 103218814 | IPO8 |  |  | 9799 | 1.035 | 0.3508 | Yes |
| 88 | 103223722 | COPA |  |  | 9930 | 1.026 | 0.3515 | Yes |
| 89 | 103243015 | SRP68 |  |  | 10052 | 1.016 | 0.3526 | Yes |
| 90 | 103239138 | ERP29 |  |  | 10138 | 1.010 | 0.3552 | Yes |
| 91 | 103242441 | LOC103242441 |  |  | 10225 | 1.006 | 0.3578 | Yes |
| 92 | 103230213 | XPO6 |  |  | 10631 | 0.982 | 0.3459 | No |
| 93 | 103242741 | AP2B1 |  |  | 11029 | 0.955 | 0.3342 | No |
| 94 | 103223285 | GGA1 |  |  | 11307 | 0.937 | 0.3277 | No |
| 95 | 103215992 | SEC24C |  |  | 11429 | 0.930 | 0.3282 | No |
| 96 | 103247047 | VPS16 |  |  | 11557 | 0.922 | 0.3284 | No |
| 97 | 103216529 | VTI1A |  |  | 11614 | 0.918 | 0.3318 | No |
| 98 | 103229212 | VTI1B |  |  | 11804 | 0.906 | 0.3291 | No |
| 99 | 103245518 | TLN2 |  |  | 11847 | 0.903 | 0.3330 | No |
| 100 | 103231443 | OXA1L |  |  | 12024 | 0.893 | 0.3308 | No |
| 101 | 103243049 | GGA3 |  |  | 12092 | 0.889 | 0.3335 | No |
| 102 | 103233984 | TNPO2 |  |  | 12213 | 0.881 | 0.3337 | No |
| 103 | 103219208 | TLN1 |  |  | 12323 | 0.874 | 0.3344 | No |
| 104 | 103231482 | AP1G2 |  |  | 12432 | 0.868 | 0.3351 | No |
| 105 | 103224962 | IPO13 |  |  | 12523 | 0.861 | 0.3366 | No |
| 106 | 103239946 | LOC103239946 |  |  | 12979 | 0.834 | 0.3215 | No |
| 107 | 103228660 | COPG1 |  |  | 13010 | 0.832 | 0.3255 | No |
| 108 | 119624280 | SCAMP3 |  |  | 13158 | 0.823 | 0.3241 | No |
| 109 | 103223009 | CLTCL1 |  |  | 13356 | 0.810 | 0.3205 | No |
| 110 | 103246067 | SCAMP5 |  |  | 13455 | 0.803 | 0.3212 | No |
| 111 | 103225827 | AGRN |  |  | 13623 | 0.794 | 0.3188 | No |
| 112 | 103218163 | RAMP1 |  |  | 14052 | 0.765 | 0.3045 | No |
| 113 | 103245324 | SCAMP2 |  |  | 14142 | 0.760 | 0.3053 | No |
| 114 | 103223888 | MTX1 |  |  | 14343 | 0.750 | 0.3011 | No |
| 115 | 103223229 | TOM1 |  |  | 14425 | 0.744 | 0.3023 | No |
| 116 | 103229082 | TOMM20L |  |  | 14569 | 0.735 | 0.3005 | No |
| 117 | 103243727 | HGS |  |  | 15045 | 0.705 | 0.2837 | No |
| 118 | 103248617 | VPS11 |  |  | 15255 | 0.692 | 0.2788 | No |
| 119 | 103223137 | AP1B1 |  |  | 15531 | 0.673 | 0.2707 | No |
| 120 | 103243189 | LOC103243189 |  |  | 15880 | 0.648 | 0.2592 | No |
| 121 | 103231094 | AP3B2 |  |  | 16105 | 0.635 | 0.2532 | No |
| 122 | 103235543 | AP2A1 |  |  | 16512 | 0.603 | 0.2389 | No |
| 123 | 103224181 | AP4B1 |  |  | 16688 | 0.593 | 0.2348 | No |
| 124 | 103245809 | VPS18 |  |  | 16953 | 0.573 | 0.2266 | No |
| 125 | 103232389 | LOC103232389 |  |  | 17277 | 0.551 | 0.2156 | No |
| 126 | 103245028 | CLTB |  |  | 17433 | 0.539 | 0.2121 | No |
| 127 | 103214724 | RRBP1 |  |  | 18175 | 0.473 | 0.1819 | No |
| 128 | 103227179 | LOC103227179 |  |  | 18380 | 0.452 | 0.1756 | No |
| 129 | 103230141 | LOC103230141 |  |  | 18982 | 0.392 | 0.1511 | No |
| 130 | 103232396 | NXF3 |  |  | 20526 | 0.185 | 0.0830 | No |
| 131 | 103244795 | CD74 |  |  | 20593 | 0.173 | 0.0812 | No |
| 132 | 103236685 | LOC103236685 |  |  | 21186 | 0.000 | 0.0546 | No |
Table: GSEA details
[plain text format]

  

Fig 2: PROTEIN\_LOCALIZATION(GO:0008104)
  
Blue-Pink O' Gram in the Space of the Analyzed GeneSet

  

Fig 3: PROTEIN\_LOCALIZATION(GO:0008104): Random ES distribution
  
Gene set null distribution of ES for
 **PROTEIN\_LOCALIZATION(GO:0008104)**
